# Efficient representation of quantum many-body states with deep neural networks

**DOI:** 10.1038/s41467-017-00705-2

**Published:** 2017-09-22

**Authors:** Xun Gao, Lu-Ming Duan

**Affiliations:** 1 Center for Quantum Information, IIIS, Tsinghua University, Beijing, 100084 China; 2 Department of Physics, University of Michigan, Ann Arbor, MI 48109 USA

## Abstract

Part of the challenge for quantum many-body problems comes from the difficulty of representing large-scale quantum states, which in general requires an exponentially large number of parameters. Neural networks provide a powerful tool to represent quantum many-body states. An important open question is what characterizes the representational power of deep and shallow neural networks, which is of fundamental interest due to the popularity of deep learning methods. Here, we give a proof that, assuming a widely believed computational complexity conjecture, a deep neural network can efficiently represent most physical states, including the ground states of many-body Hamiltonians and states generated by quantum dynamics, while a shallow network representation with a restricted Boltzmann machine cannot efficiently represent some of those states.

## Abstract (web summary)

One of the challenges in studies of quantum many-body physics is finding an efficient way to record the large system wavefunctions. Here the authors present an analysis of the capabilities of recently-proposed neural network representations for storing physically accessible quantum states.

## Introduction

The Hilbert space dimension associated with quantum many-body problems is exponentially large, which poses a big challenge for solving those problems even with the most powerful computers. The variational approach is usually the tool of choice for tackling such difficult problems, which include many successful examples from simple mean-field approximation to more complicated methods such as those based on matrix product states^1^, tensor network states^2^, string bond states^3, 4^, and, more recently, neural network states^5, 6^. The first important step of the variational approach is to find an efficient representation of the relevant quantum many-body states. Here, by efficient we mean the number of parameters used to characterize those quantum states increases at most by a polynomial function with the number of particles (or degrees of freedom) in the system. With an efficient representation, one can combine it with powerful learning methods to optimize those variational parameters by optimization techniques, such as the gradient descent method.

Neural networks are a powerful tool to represent complex correlations in multiple-variable functions or probability distributions and recently find wide applications in artificial intelligence through the popularity of deep learning methods^7^. An interesting connection has been made recently between the variational approach in quantum many-body problems and learning methods based on neural network representations^5^. Numerical evidence suggests that the restricted Boltzmann machine (RBM), a shallow generative neural network, optimized by the reinforcement learning method, provides a good solution to several many-body models^5^. Given this success, an important open question is what characterizes the representational power and limitations of the RBM for quantum many-body states.

In this paper, we characterize the representational power and limitations of the RBM and its extension to deep neural networks, the deep Boltzmann machine (DBM). We prove that DBMs can efficiently represent most physical states, including the ground states of many-body Hamiltonians and states generated by quantum dynamics, while RBMs cannot efficiently represent some of those states. The result shows there exists an exponential separation in efficiency between using DBMs or RBMs to represent quantum many-body states.

## Results

### Summary of major results

Our first major result concerns RBMs. We prove that while RBMs can efficiently represent many highly entangled states, there is a fundamental limit for them to efficiently represent general quantum states. For the power of RBMs, we show through explicit construction that RBMs can efficiently and exactly represent arbitrary graph states^8^, certain states obeying entanglement volume law or describing the critical system^9^, and topological toric code states^10^. For the limitation of RBMs, we introduce an explicit class of states which can be generated either by a polynomial-size quantum circuit or as ground states of gapped Hamiltonians, and prove for those states there is no efficient RBM representation unless the polynomial hierarchy, a generalization of the famous P versus NP problem in computer science, collapses, which is widely believed to be unlikely. Note that our result well complements the known theory about the representational power of RBMs^11, 12^. It has been proven in ref. ^12^ that an RBM can approximate any probability distribution with arbitrary accuracy if one does not limit the representation efficiency (the number of parameters in the representation). Here, we strengthen this result by showing that with consideration of efficiency, there are quantum probability distributions that cannot be efficiently represented by RBMs.

Our second major result concerns about the power of DBMs. We prove through explicit construction that DBMs can efficiently represent any states generated by polynomial-size quantum circuits or any ground states of physical Hamiltonians with polynomial-size gaps. Here, polynomial-size gap means that the energy gap of the Hamiltonian approaches to zero at most by 1/poly(*n*), where poly(*n*) denotes a polynomial function of the particle number *n*. Most physical quantum states are generated either by many-body dynamics, which can be efficiently simulated through a polynomial-size quantum circuit^13–15^, or as ground states of some physical Hamiltonians, so they can all be efficiently represented by DBMs. This result, combined with the reinforcement learning method (see discussion in Supplementary Note 6), indicates the potential power of the DBM representation as a tool for solving quantum many-body problems.

We note that existence of an efficient representation by a DBM does not mean we can always use this representation for efficient calculation of physical observables as the latter involves further complicated index contraction. Efficient representation is a necessary but not sufficient condition required to tackle quantum many-body problems. One need to combine it with efficient numerical training algorithm to extract physical observables. Finding ground-state energies of general many-body Hamiltonians is known to be computationally hard, requiring in general exponential calculation time^2^. So even if an efficient representation of ground states exists, we may not be able to use it to find ground-state energy. On the other hand, although we prove that the RBMs cannot represent the most general quantum states, it does not restrict the use of RBMs for solving many practical problems. Indeed, RBMs could be very useful to represent and learn a wide class of ground states or physical states arising from time evolution. Apart from numerical simulation of quantum many-body problems, efficient representation by DBMs or RBMs may also find applications for the classification of topological quantum phases^16, 17^ or the quantum approach to space–time with holographic properties^18, 19^, similar to applications of the tensor network representation in those scenarios.

### Neural network quantum states

**Fig. 1:**
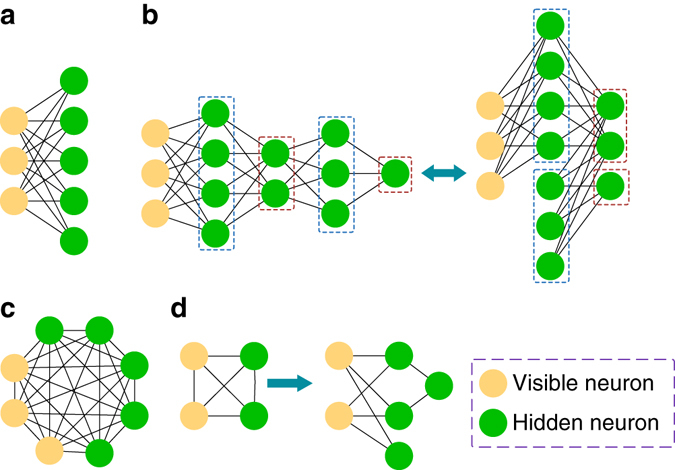
Illustration of Boltzmann machine neural networks. **a** Restricted Boltzmann machine (RBM) which has only one hidden layer and no intra-layer connections. **b** Deep Boltzmann machine (DBM) which has at least two hidden layers and no intra-layer connections. General DBMs are equivalent to DBMs with two hidden layers after rearrangement of odd and even layers. **c** Fully connected Boltzmann machine which has intra-layer connections. **d** Reduction of fully connected Boltzmann machine to DBMs with two hidden layers

A many-body quantum state of *n* qubits can be written as $\left| \Psi \right\rangle = \mathop {\sum}\nolimits_{\bf{v}} {\Psi \left( {\bf{v}} \right)\left| {\bf{v}} \right\rangle }$ in the computational basis with **v** ≡ (*v*_1_, …, *v*_*n*_), where the wave function Ψ(**v**) is a general complex function of *n* binary variables *v*_*i*_ ∈ {0, 1}. In the neural network representation by a Boltzmann machine, the wave function Ψ(**v**) is expressed as $\Psi ({\bf{v}}) = \mathop {\sum}\nolimits_{\bf{h}} {{{\rm{e}}^{W({\bf{v}},{\bf{h}})}}}$, where the weight *W*(**v**, **h**) is a complex quadratic function of binary variables **v** and **h** ≡ (*h*_1_, …, *h*_*m*_) called visible and hidden neurons, respectively. The number of hidden neurons *m* is at most poly(*n*) for an efficient representation. In the graphic representation shown in Fig. 1, the neurons *v*_*i*_ and *h*_*j*_ connected by an edge are correlated with a nonzero *W*_*ij*_ in the weight $W({\bf{v}},{\bf{h}}) = \mathop {\sum}\nolimits_{i,j} {{W_{ij}}{v_i}{h_j}}$. For the RBM (Fig. 1a), the layer of visible neurons is connected to one layer of hidden neurons (neurons in the same layer are not mutually connected). The DBM is similar to the RBM but with two or more layers of hidden neurons (Fig. 1b). Two hidden layers are actually general enough as one can see in Fig. 1b that odd and even layers can each be combined into a single layer. A fully connected Boltzmann machine is shown in Fig. 1c. In the methods section, we prove that any fully connected Boltzmann machine can be efficiently represented by a DBM as illustrated in Fig. 1d.

### Power and limitations of restricted Boltzmann machines

**Fig. 2:**
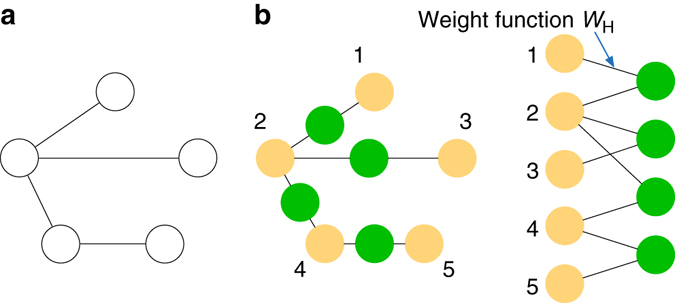
Representation of Graph states by RBMs. **a** Graph representation of an example graph state. **b** Representation of the graph state with a restricted Boltzmann machine. One hidden neuron with the Hadamard weight function *W*_H_ (explicit form given in Eq. (1) of the text) simulates the correlation in the wave function between each pair of connected qubits in any graph states

First, we show that RBMs can represent many highly entangled states, including wave functions of any graph states^8^, topological toric codes^10^, and states violating the entanglement area law or describing the critical system^9^. As an example to illustrate the method, we give a simple construction for RBM representation of any graph state and leave the representation of other categories of states to Supplementary Note 1. RBM representations for one-dimensional cluster states (a special case of graph states) and toric codes have been given recently in ref. ^6^. We give a different construction method which is simpler and more systematic. The wave function of a graph state takes the form $\Psi ( {{v_1}, \cdots ,{v_n}} ) = \mathop {\prod}\nolimits_{\langle {i,j} \rangle } {{{( - 1)}^{{v_i}{v_j}}}{\rm{/}}\sqrt 2 }$, where 〈*i*, *j*〉 denotes an edge linking the *i*-th and *j*-th qubits represented by visible neurons *v*_*i*_, *v*_*j*_. As shown in Fig. 2, one hidden neuron *h* and two edges with weight *W*_H_ realize the correlation function ${( - 1)^{{v_i}{v_j}}}{\rm{/}}\sqrt 2$ between *v*_*i*_ and *v*_*j*_. This requires solving the equation $\mathop {\sum}\nolimits_h {{{\rm{e}}^{{W_{\rm{H}}}( {{v_i},h} ) + {W_{\rm{H}}}( {{v_j},h} )}} = {{( { - 1} )}^{{v_i}{v_j}}}{\rm{/}}\sqrt 2 }$, which has a simple solution

1 $${W_{\rm{H}}}(x,h) = \frac{\pi }{8}i - \frac{{{\rm{ln}}\,2}}{2} - \frac{\pi }{2}ix - \frac{\pi }{4}ih + i\pi xh$$

with *x* = *v*_*i*_ or *v*_*j*_.

The RBM state has an important property that its wave function Ψ(**v**) can be calculated efficiently under given input values to the variables *v*_*i*_. Here we prove that this property leads to limitations of the RBM in representing more general quantum states. With a given input value of **v**, Ψ(**v**) can be factorized as

2 $$\mathop {\prod}\limits_j {\left( {\mathop {\prod}\limits_{i:\left\langle {i,j} \right\rangle } {{{\rm{e}}^{{W_{ij}}\left( {{v_i},0} \right)}}} + \mathop {\prod}\limits_{i:\left\langle {i,j} \right\rangle } {{{\rm{e}}^{{W_{ij}}\left( {{v_i},1} \right)}}} } \right)} ,$$

where *i* (*j*) runs from 1 to at most *n* (*m*), so the total computational time for Ψ(**v**) scales as *mn* for each given input **v**. This means Ψ(**v**) can be computed by a circuit *C*_*n*_ with polynomial size poly(*n*) for a given input **v** ∈ {0, 1}^*n*^. If a quantum state has a RBM representation (even if its explicit form is unknown), computing Ψ(**v**) is characterized by the computational complexity class P/poly^20^, which represent problems that can be solved by a polynomial-size circuit even if the circuit cannot be constructed efficiently in general. The circuit here corresponds to a RBM representation, with the input given by a specific **v** and the output given by the value of Ψ(**v**).

We have introduced in ref. ^21^ a specific quantum many-body state, denoted as Ψ_GWD_, for which we proved it is #P-hard to calculate its wave function Ψ_GWD_(**v**) in the computational basis **v** (#P-hard is a known computational complexity class that in general requires exponential calculation time). If this state Ψ_GWD_ has a RBM representation, it means #P ⊂ P/poly, an unlikely result in computational complexity theory as this means the polynomial hierarchy collapses^22^. The state Ψ_GWD_ (with its explicit form given in Supplementary Note 3) is just a two-dimensional cluster state after a layer of translation-invariant single-qubit unitary operations. This state Ψ_GWD_ is a special instance of states that can be generated by a constant-depth quantum circuit (which is a special polynomial-size circuit). It also belongs to the projected entangled pair states (PEPS) and the ground states of gapped Hamiltonians. Combining the results above, we arrive at the following theorem:

Theorem 1: There exist states, which can be generated by a constant-depth quantum circuit or expressed as PEPS or ground states of gapped Hamiltonians, but cannot be efficiently represented by any RBM unless the polynomial hierarchy collapses in the computational complexity theory.

The above argument holds for the exact representation of Ψ(**v**) with an RBM. As proved in Supplementary Note 3, under reasonable conjectures about computational complexities, the same result also holds for approximate representations of Ψ(**v**) with RBMs.

Note that 2D cluster states can be efficiently represented by RBMs. While after a layer of single-qubit operations which do not change the quantum phase according to the classification scheme in refs. ^16, 23^, the output state Ψ_GWD_ cannot be efficiently represented by RBMs any more. So the RBM representation is not closed under unitaries that preserve a quantum phase.

### Representational power of deep Boltzmann machines

Now we show with DBMs, i.e., with one more layer of hidden neurons, most physical states, including all the states in Theorem 1, can be efficiently represented. For this purpose, first we introduce a couple of gadgets that will simplify our construction.

**Fig. 3:**
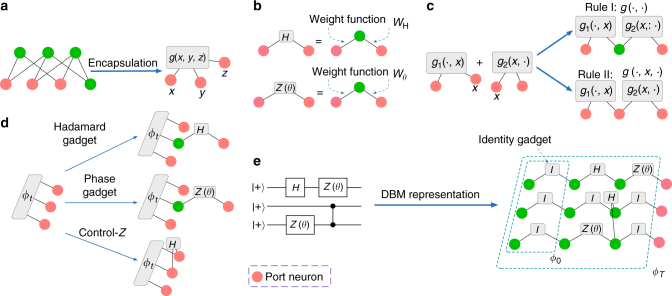
Representation of universal quantum computational states by DBMs. **a** Gadget is a complex function of binary variables represented by port neurons, a short-hand notation after encapsulation of hidden neurons. **b** Two elementary gadgets for representation of quantum circuits: the Hadamard gadget with weight *W*_H_ given by Eq. (1) and the phase gadget with weight *W*_*θ*_ given by Eq. (3). **c** Two types of fusion rules for gadgets: rule I and rule II and their neural network representation. **d** Fusion with Hadamard or phase gadgets with rule I or rule II simulates application of three elementary quantum gates: the Hadamard gate, the phase gate, and the controlled phase flip gate, which together make universal quantum computation. The figure illustrates evolution of the wave function from step *t* to step *t* + 1. **e** Representation of an example quantum circuits with elementary gadgets. To represent circuits of depths *T*, we need to apply *T* steps of fusions with elementary gadgets, and gadget fusions in the same step can be applied in parallel. The identity gadget is a special phase gadget with *θ* = 0. After the last step of computation, port neurons become visible neurons to represent the index of physical qubits, and we get a DBM representation of the output state

A gadget is a complex function of binary variables after encapsulation of hidden neurons in a DBM network as shown in Fig. 3a, where the input is represented by port neurons (for connection of different gadgets) and the output is the value of the function. We use gadgets as basic elements in a large DBM. As examples, we define the Hadamard gadget and phase gadget as shown in Fig. 3b, which will play the role of elementary gates for construction of DBM representations of quantum circuits. The weight function *W*_H_ is given by Eq. (1) and *W*_*θ*_ is the solution of the equation $\mathop {\sum}\nolimits_h {{{\rm{e}}^{{W_\theta }\left( {{x_1},h} \right) + {W_\theta }\left( {{x_2},h} \right)}} = {{\rm{e}}^{i\theta {x_1}}}{\delta _{{x_1}{x_2}}}}$, which may take the form

3 $${W_\theta }(x,h) = - \frac{{{\rm{ln}}\,2}}{2} + \frac{\theta }{2}ix + i\pi xh.$$

We can combine two gadgets *g*_1_, *g*_2_ into one gadget *g* by two types of fusion rules shown in Fig. 3c:

4 $${\rm{rule}}\,{\rm{I}}\!\!:g( \cdot , \cdot ) = \mathop {\sum}\limits_x {{g_1}( \cdot ,x){g_2}(x, \cdot )} ,$$

5 $${\rm{rule}}\,{\rm{II}}\!\!:g( \cdot ,x, \cdot ) = {g_1}( \cdot ,x){g_2}(x, \cdot ),$$

where rule I simulates matrix multiplication.

With these tools, now we construct efficient DBM representations of any quantum states generated by a polynomial-size circuit. The Hadamard gadget and phase gadget shown in Fig. 3b are used to construct three elementary quantum gates: Hadamard gate *H*, phase gate *Z*(*θ*) with an arbitrary phase *θ*, and controlled phase flip gate CZ, which together are universal for quantum computation^24, 25^. The initial state of the circuit is taken as (|0〉 + |1〉)^⊗*n*^, an equal superposition of computational basis states, which is represented by the wave function *ϕ*_0_(*x*_1_, …, *x*_*n*_) = 1, the identity gadget. Denote the wave function after applying *t*-layer of elementary gates as *ϕ*_*t*_(*x*_1_,…, *x*_*n*_). As shown in Fig. 3d, using rule I (corresponding to matrix multiplication), the Hadamard gadget and phase gadget simulate gates *H* and *Z*(*θ*). Using rule II with the Hadamard gadget, we have

6 $${\phi _{t + 1}}\left( { \cdots {x_i},{x_{i + 1}} \cdots } \right) = {\left( { - 1} \right)^{{x_i}{x_{i + 1}}}}{\phi _t}\left( { \cdots {x_i},{x_{i + 1}}, \cdots } \right){\rm{/}}\sqrt 2 ,$$

which simulates the CZ gate except for the unimportant normalization factor 1/$\sqrt 2$. The above procedure can be parallelized as illustrated in Fig. 3e, which shows the DBM representation of an example circuit. For a quantum circuit of depth *T*, we apply *T* steps of fusion rules, and each step needs *O*(*n*) neurons. So the DBM representation of the output state of the quantum circuit takes *O*(*nT*) neurons. This DBM representation is sparse, meaning that each neuron has a constant coordination number (number of connected edges) that does not increase with the size of neural network. We therefore have the following theorem:

Theorem 2: Any quantum state of *n* qubits generated by a quantum circuit of depth *T* can be represented exactly by a sparse DBM with *O*(*nT*) neurons.

**Fig. 4:**
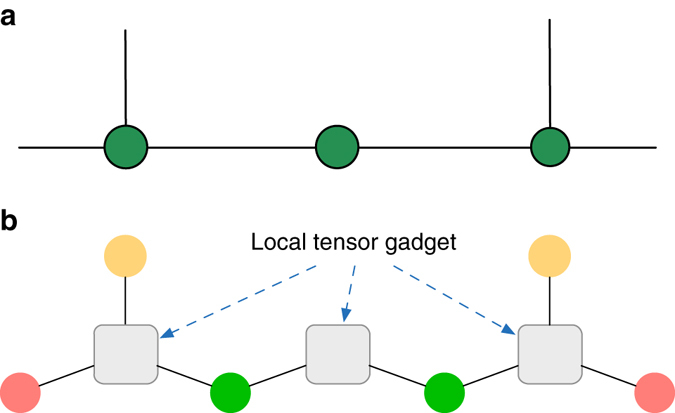
Representation of tensor network states with DBMs. **a** Illustration of a tensor network state. **b** Representation of this tensor network state by a DBM. Visible (hidden) neurons play the role of physical (bond) indices, respectively. Port neuron represents either the bond index for the next step of tensor contraction or the physical index if there is no further contraction. The *gray box* stands for the local tensor gadget ${A_{{x_1} \cdots {x_c}}}$ which can be efficiently represented with a DBM

Using the above theorem, we now construct efficient DBM representation of any tensor network states, which include the PEPS and the multi-scale entanglement renomalization ansatz (MERA) as special cases^26, 27^. Suppose the local tensor is ${A_{{b_1} \cdots {b_d}p}}$, which has one (or zero) physical index *p* and *d* bond indices *b*_1_, …, *b*_*d*_, each ranging from 1 to the bond dimension *D*. Without loss of generality, we assume *p* is binary and *D* = 2^*k*^ for some integer *k* and write the local tensor as a function ${A_{{x_1} \cdots {x_c}}}$, where each *x*_*i*_ is a binary variable and *c* = *kd* + 1. The state of $\left| A \right\rangle = \mathop {\sum}\nolimits_{{x_1}, \cdots ,{x_c}} {{A_{{x_1} \cdots {x_c}}}\left| {{x_1}, \cdots ,{x_c}} \right\rangle }$ can be generated by a quantum circuit with the number of elementary gates on the order of *O*(2^2(*kd*+1)^) = *O*(*D* ^2*d*^)^25^, which is square of the Hilbert space dimension of span (|*x*_1_, …, *x*_*c*_〉). Using Theorem 2, the state |*A*〉 can be represented exactly by a DBM with *O*(*D* ^2*d*^) neurons, and the resultant representation is called the local tensor gadget. We use fusion rule I to link two local tensor gadgets to simulate contraction of bond index and put physical index in the visible layer, as shown in Fig. 4. We thus have the following theorem:

Theorem 3 A tensor network state with bond dimension *D*, maximum coordination number *d*, and *n* local tensors, can be represented efficiently and exactly by a sparse DBM with *O*(*nD* ^2*d*^) neurons.

We use Theorem 3 as a tool to prove the following major Theorem 4 for the representation of ground states of physical Hamiltonians, and for this proof we take *D* = 2 and *d* = 4, so the factor *O*(*D* ^2*d*^) becomes a moderate constant in the representation. The detailed proof of Theorem 4 is included in Supplementary Note 5. The basic idea is to use tensor network state to simulate Hamiltonian evolution along imaginary time until one arbitrarily approaches the ground state^28, 29^. Recently, quantum simulation based on truncated Taylor series has been proposed^15^ which has exponential improvement on precision compared to traditional methods based on Trotter decomposition. Inspired by this idea, we construct tensor network simulation for imaginary time evolution of any Hamiltonian based on truncated Taylor series. Compared to the previous method^28^, our construction offers exponential improvement on precision in representation. We arrive at the following theorem:

Theorem 4 The ground state of any Hamiltonian can be represented by a sparse DBM with neuron number

7 $$O\left( {\frac{1}{{\it{\Delta}} }\left( {n + {\rm{log}}\frac{1}{\epsilon }} \right){m^2}} \right),$$

where *n* is the particle number, *m* is the number of interaction terms in the Hamiltonian, *Δ* is the energy gap, and *ε* is the representational error.

This representation is efficient as long as the energy gap *Δ* vanishes with size of the system *n* no faster than 1/poly(*n*), which is typically true for physical Hamiltonians (even if they are gapless in the thermodynamic limit).

## Discussion

With the success of deep learning methods, a question often raised is why the depth of a neural network is so important^7^. Our proof of the exponential separation in efficiencies of using DBMs and RBMs to represent quantum many-body states helps to address this question in the context of the quantum world. We have proven that most physical states, either from quantum dynamics or as ground states of many-body Hamiltonians, can be efficiently represented by DBMs. Our proof provides a systematic approach to construct polynomial-size DBMs to represent those quantum states. In practice, one typically use learning algorithms to directly find RBMs or DBMs to approximate the quantum states, and in that case, depending on the problem, significantly smaller size neural networks could be found compared with what is indicated by those general theorems. In Supplementary Note 6, we introduce a prototype reinforcement learning algorithm to train DBMs to approximate ground states. Confronted with real strongly correlated models, there is certainly much room for improving the learning algorithms with either RBMs or DBMs, which represents an interesting frontier for computational many-body physics. The rigorous results obtained in this paper, on the one hand, show the power and limitations of representing quantum states with RBMs or DBMs, which is a question of fundamental interest, and on the other hand, may find interesting applications in different areas that require efficient representation of quantum states, including, for instance, classification of topological quantum phases^16, 17^, construction of holographic models^18, 19^, and solving quantum many-body problems^5^.

## Methods

### Representation of a fully connected Boltzmann machine by a DBM

Here we prove that any fully connected Boltzmann machine (with intra-layer edges) can be efficiently simulated with DBMs (without intra-layer connections) as shown in Fig. 1d. The key point is to simulate the interaction between two neurons by a gadget $\mathop {\sum}\nolimits_h {{{\rm{e}}^{{W_1}\left( {{x_1},h} \right) + {W_2}\left( {{x_2},h} \right)}} = {{\rm{e}}^{{W_0}\left( {{x_1},{x_2}} \right)}}}$. Suppose the interaction term is *Jx*_1_ *x*_2_ in *W*_0_, we need *W*_1_ + *W*_2_ = *a* − ln 2 + *b*(*x*_1_ + *x*_2_)(2*h* − 1) + *c*(2*h* − 1) + *d*(*x*_1_ + *x*_2_) to simulate the interaction with the aid of hidden neuron *h*, where the parameters *a*, *b*, *c*, *d* need to satisfy the equations e^*a*^ cosh(*c*) = 1, e^*a*^e^*d*^ cosh(*b* + *c*) = 1, e^*a*^e^2*d*^ cosh(2*b* + *c*) = e^*J*^. These equations have solutions, one of them is

8 $$a = - d = - J{\rm{/}}2,b = - c = - i\,{\rm{arccos}}( {{{\rm{e}}^{J/2}}} ).$$

### Derivation of the weight functions *W*_H_ and *W*_*θ*_

In the main text, we give the expression for *W*_H_ and *W*_*θ*_ which can be obtained by setting a general form for them as *a* + *bx* + *ch* + *dxh* and solving the resultant equations for the parameters *a*, *b*, *c*, *d*. Here, we give the detailed derivation.

For the Hadamard gadget which is used to construct RBM representation for graph states and simulate *H* and CZ gates, the equation we need to solve is

9 $$\mathop {\sum}\limits_{h = 0,1} {{{\rm{e}}^{{W_{\rm{H}}}\left( {{x_{1,}}h} \right) + {W_{\rm{H}}}\left( {{x_{2,}}h} \right)}} = {H_{{x_1}{x_2}}}} ,$$

where the correlation

10 $${H_{{x_1}{x_2}}} = \frac{{{{( - 1)}^{{x_1}{x_2}}}}}{{\sqrt 2 }} = {\rm{cos}}\left( {\frac{\pi }{4}\left[ {2\left( {{x_1} + {x_2}} \right) - 1} \right]} \right).$$

The last step in Eq. (10) is valid since we have ${H_{{x_1}{x_2}}} = 1{\rm{/}}\sqrt 2$, 1/$\sqrt 2$, −1/$\sqrt 2$ when *x*_1_ + *x*_2_ = 0, 1, 2, respectively. Using the relation

11 $${\rm{cos}}\,X = \frac{{{{\rm{e}}^{iX}} + {{\rm{e}}^{ - iX}}}}{2} = \mathop {\sum}\limits_h {{{\rm{e}}^{iX(2h - 1) - {\rm{ln}}\,2}}} ,$$

with *X* = *π*/4[2(*x*_1_ + *x*_2_) − 1], we get

$${W_{\rm{H}}}\left( {{x_{i,}}h} \right) = i\pi {x_i}h - i\pi \left[ {2{x_i} + h} \right]{\rm{/}}4 + \left( {i\pi {\rm{/}}4 - {\rm{ln}}\,2} \right){\rm{/}}2,$$

for *x*_*i*_ = *x*_1_ or *x*_2_.

For the phase gadget which is used to simulate *Z*(*θ*) gate, it needs to satisfy

12 $$\mathop {\sum}\limits_{h = 0,1} {{{\rm{e}}^{{W_\theta }\left( {{x_{1,}}h} \right) + {W_\theta }\left( {{x_{2,}}h} \right)}} = Z{{(\theta )}_{{x_1}{x_2}}} = {\delta _{{x_1}{x_2}}}{{\rm{e}}^{i\theta {x_1}}}} .$$

We simulate ${\delta _{{x_1}{x_2}}}$ by the following observation:

13 $${\delta _{{x_1}{x_2}}} = \frac{{1 + {{\rm{e}}^{i\pi \left( {{x_1} + {x_2}} \right)}}}}{2} = \mathop {\sum}\limits_h {{\rm{e}}^{i\pi \left( {{x_1} + {x_2}} \right)h - {\rm{ln}}\,2}}.$$

Note that ${\delta _{{x_1}{x_2}}}{{\rm{e}}^{i\theta {x_1}}} = {\delta _{{x_1}{x_2}}}{{\rm{e}}^{i\theta \left( {{x_1} + {x_2}} \right)/2}}$, we have the solution

14 $${W_\theta }\left( {{x_{i,}}h} \right) = i\pi {x_i}h + \left( {i\theta {x_i} - {\rm{ln}}\,2} \right){\rm{/}}2.$$

### Data availability

The data that support the findings of this study are available from the corresponding author upon request.

## Electronic supplementary material

Supplementary Information
