# Supplementary material for: Efficient representation of quantum many-body states with deep neural networks
Source: Nat Commun. 2017 Sep 22;8:662. doi: 10.1038/s41467-017-00705-2 (PMC5610197; doi:10.1038/s41467-017-00705-2)
Supplement: Supplementary file 1 — Supplementary Information [file 41467_2017_705_MOESM1_ESM.pdf]

Description of Supplementary Files

File Name: Supplementary Information
Description: Supplementary Figures, Supplementary Notes and Supplementary References

Supplementary Note 1

RBM representation of many-body entangled states

In this construction, we restrict to simple RBM gadget with only one hidden neuron connected to k visible neurons and identical weight function W on each edge. we need to solve the equation

$$\sum_h e^{W(v_1,h)+\cdots+W(v_k,h)} = g(v_1,\cdots,v_k) \tag{1}$$

for a certain correlation $g(v_1,\cdots,v_k)$. Apart from the graph state example given in the main text, we construct RBM representation of three classes of entangled states: the toric code, which is the simplest state with topological order and useful for quantum error correction; the randomly distributed entangled pair state, which satisfies the entanglement volume law instead of the area law [1]; and the coherent thermal state which describes a critical system [2]. These examples and the corresponding RBM representations are shown in Supplementary Fig. 1.

For the toric code, the correlation function $g(v_1,v_2,v_3,v_4) = (v_1+v_2+v_3+v_4 \mod 2)$ on each vertex and the wave function is a product of these functions over all vertices [3]. We have

$$g(v_1,v_2,v_3,v_4) = \frac{1+e^{i\pi(v_1+v_2+v_3+v_4)}}{2} = \sum_{h=0,1} e^{i\pi(v_1+v_2+v_3+v_4)h-\ln 2}, \tag{2}$$

so the weight $W(v_i,h) = i\pi v_i h - (\ln 2)/4$ for the toric code example. Supplementary Ref.[4] gives another construction of RBM representation of the toric code state, and compared with that our construction here is significantly simpler.

For the randomly distributed entangled pair states, we have $g(v_1,v_2) = \delta_{v_1 v_2}$ for each entangled pair $|00\rangle + |11\rangle$. The weight function $W(v_i,h)$ is a special case of the phase gadget W_θ with $\theta = 0$ (the identity gadget).

For a coherent thermal state defined as

$$|\Psi_{ch}\rangle = \sum_{\mathbf{v}} \prod_{\langle i,j\rangle} e^{-\beta v_i v_j/2} |\mathbf{v}\rangle, \tag{3}$$

it has the same correlation function as the corresponding thermal state with β denoting the inverse temperature [2]. We consider the binary variables $\mathbf{v}$ defined on a square lattice as shown in Supplementary Fig. 1, and the state $|\Psi_{ch}\rangle$ then defines a coherent thermal state for a 2D Ising model which has a phase transition at $\beta = \beta_c$. At this critical point β_c, the state $|\Psi_{ch}\rangle$ describes a many-body entangled state for a critical system. For any value of β, the wave function of the state $|\Psi_{ch}\rangle$ can be simply represented by a RBM as shown in Supplementary Fig. 1. We have $g(v_1,v_2) = e^{-\beta v_1 v_2/2}$ for each pair of visible neurons. The correlation is identical to the case that we have considered for the DBM representation of a fully connect Boltzmann machine (see the method section of the main text). We just need a single hidden neuron to generate this correlation with the weight function given in the method section of the main text where J is replaced by $-\beta/2$.

Supplementary Note 2

A review of definition of related complexity classes

For completeness, here we review some definitions of complexity classes related to our proof for the limitation of RBM representation. More detailed discussion can be found in Supplementary Ref. [5]. The concept of language L (a subset of the string $\{0,1\}^*$) is used to formalize decision problems (of which solution can only be true or false). We call an instance of the problem as x; if the solution of x is true, $x \in L$, otherwise $x \notin L$.

First, we review the definitions of the complexity classes P, NP, and the polynomial hierarchy:

Definition 1 (P) A language L is in *P* if there exists a polynomial time classical Turing Machine M such that for every $x \in \{0,1\}^*$

$$x \in L \Leftrightarrow M(x) = 1.$$

This class characterizes problems which can be solved efficiently by classical computer without randomness. In contrast, NP-hard is usually used to characterize difficult problems. Intuitively, NP is the set of problems for which the “yes” solutions can be efficiently verified by a classical computer.

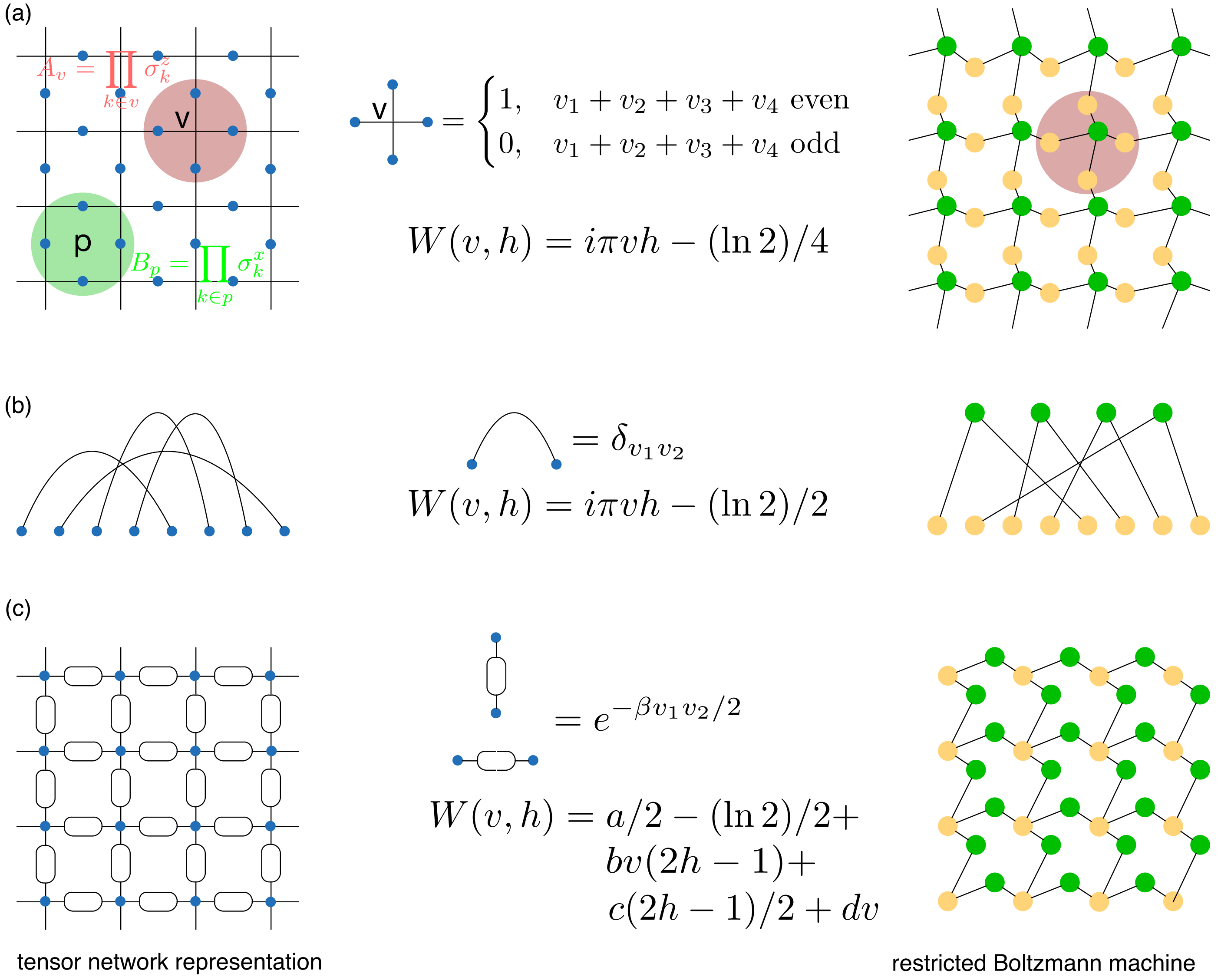

Supplementary Fig. 1: **RBM representation of some many-body entangled states. a,** Toric code is the simplest topologically ordered state used as a quantum error correcting code, which is defined as the stabilizer state of stabilizers A_v and B_p, where v and p denote vertex and plaquette, respectively. The wave function for the toric code is a product of functions shown in the figure for each vertex v and there is no constraint on plaquette p. **b,** Randomly distributed entangled pairs $|00\rangle + |11\rangle$ on a line (or any lattice). The state obeys the entanglement volume law. The wave function of this state is a simple product of functions shown in the figure for each pair. **c,** Coherent thermal state which represents a critical system when β reaches β_c, the critical point in the corresponding thermal state of the classical Ising model. The wave function is square root of the corresponding probability in classical thermal model. For RBM representation, the coefficient a, b, c, d is given by the solution in the method section of the main text with J replaced by $-\beta/2$.

Definition 2 (NP: nondeterministic polynomial) A language L is in NP if there exists a polynomial p and a polynomial time classical Turing Machine M such that for every $x \in \{0,1\}^*$

$$x \in L \Leftrightarrow \exists u \in \{0,1\}^{p(|x|)} \text{ s.t. } M(x,u) = 1.$$

Polynomial hierarchy is a generalization of the complexity class NP. We call u a witness.

Definition 3 (Σ_i^p,PH: polynomial hierarchy) For $i \geq 1$, a language L is in Σ_i^p if there exists a polynomial q and a polynomial time classical Turing Machine M such that for every $x \in \{0,1\}^*$

$$\begin{aligned} x \in L \quad \Leftrightarrow \quad & \exists u_1 \in \{0,1\}^{q(|x|)} \forall u_2 \in \{0,1\}^{q(|x|)} \cdots Q_i u_i \\ & \in \{0,1\}^{q(|x|)} \text{ s.t. } M(x, u_1, u_2, \cdots, u_i) = 1, \end{aligned}$$

where Q_i denotes $\forall$ or $\exists$ depending on whether i is even or odd, respectively. And

$$\mathsf{PH} = \bigcup_i \Sigma_i^p.$$

Note that $\mathsf{NP} = \Sigma_1^p$ and one can extend i to 0 such that $\mathsf{P} = \Sigma_0^p$. Clearly, $\Sigma_i^p \subseteq \Sigma_{i+1}^p \subseteq \mathsf{PH}$. Most computer scientists believe $\mathsf{P} \neq \mathsf{NP}$. A generalization of this conjecture is that for every i, Σ_i^p is strictly contained in Σ_{i+1}^p, which means $\Sigma_i^p \neq \Sigma_{i+1}^p$. It can also be stated as "the polynomial hierarchy does not collapse". This conjecture is often used in complexity theory.

We also used the complexity classes P/poly and #P in proof of theorem 1. Boolean circuit model is often used in computational complexity theory in addition to the Turing machine. The complexity class P/poly characterizes those problems that can be solved by polynomial size boolean circuits.

Definition 4 (P/poly) Polynomial-sized circuit family is a sequence of $\{C_n\}$ of boolean circuit, where the input size of C_n is n and the size of C_n is bounded by a poly(n). P/poly is the class of languages which can be decided by polynomial-sized circuit family.

The difference between P/poly and P is that the circuit may not be built using an efficient Turing machine.

Another way to generalize the complexity class NP is to go to the so-called counting problem. According to the definition of NP, it only requires knowing whether there exists at least one witness such that the Turing machine accepts. We can generalize NP to counting the number of witnesses that the Turing machine accepts. This complexity class is defined as #P

Definition 5 (#P) A function f is in $\#P$ if there exists a polynomial q and a polynomial time classical Turing machine M such that for every $x \in \{0,1\}^*$

$$f(x) = \#\{y \in \{0,1\}^{q(|x|)} : M(x,y) = 1\}.$$

Different from other complexity classes defined for decision problems, #P defines problems of which output is not 0 or 1, but an integer number. So in order to compare decision problems and counting problems, it is convenient to introduce oracle machine: $\mathsf{P}^{\mathcal{O}}$ is the problem decided by a polynomial deterministic Turing machine with access to a sub-routine or oracle $\mathcal{O}$ (which is not necessarily efficiently computable, but we only count the number of times required to access the oracle.) For example, P^{NP} represents the set of problems that can be decided by a polynomial Turing machine with a polynomial number of times to access a sub-routine which can solve NP problems.

Supplementary Note 3

Proof of theorem 1 under approximation representation

Here we introduce a specific state on 2D lattice, denoted as $|\psi_{\mathrm{GWD}}\rangle$ and shown in Supplementary Fig. 2. State $|\psi_{\mathrm{GWD}}\rangle$ is cluster state after one layer translation-invariant single-qubit unitary transformation. This state was introduced in Supplementary Ref. [6] for proof of quantum supremacy. We prove here $|\psi_{\mathrm{GWD}}\rangle$ cannot be efficiently represented by RBMs under reasonable conjectures in complexity theory. This no-go theorem holds for both exact and approximate representation.

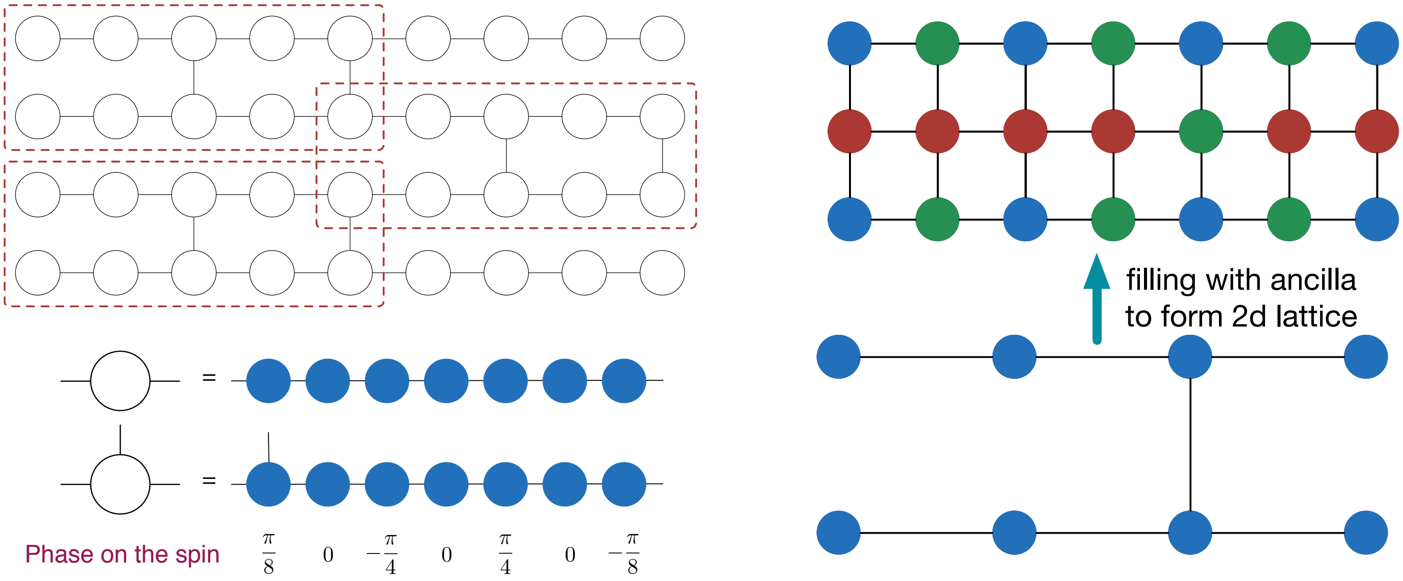

Supplementary Fig. 2: **Construction of a state that cannot be represented by a RBM.** The state $|\psi_{\mathrm{GWD}}\rangle$ used in the proof of theorem 1, is introduced in Supplementary Ref. [6] for proof of quantum Supremacy. To construct this state, we start from a brickwork of white circles [7] (the left top side), with each white circle represented by seven blue circles. Each blue circle represents a qubit, and the brickwork of blue circles can be filled with additional red and green circles (each represent an ancillary qubit) to form a regular 2D square lattice (shown on the right top side). We start with a standard 2D cluster state for the square lattice, and then apply the phase gates $Z(\theta)$ on the blue-circle qubits with the angle θ forming a periodic pattern shown in the left bottom figure and the Hadamard gate on each green-circle and blue-circle qubits (no gate on the red-circle qubits). After this layer of single-qubit unitary operations, we get the state $|\psi_{\mathrm{GWD}}\rangle$.

Exact representation

Suppose the coefficient of $|\psi_{\mathrm{GWD}}\rangle$ in the computational basis is $\Psi(\mathbf{v})$ (In term of notation, $|\Psi(\mathbf{v})|^2$ corresponds to q_x in Supplementary Ref. [6]). In Supplementary Ref. [6], it is proved that computing $|\widetilde{\Psi}(\mathbf{v})|^2$ is #P-hard where $|\widetilde{\Psi}(\mathbf{v})|^2$ is an estimation of $|\Psi(\mathbf{v})|^2$ such that

$$\left||\Psi(\mathbf{v})|^2 - |\widetilde{\Psi}(\mathbf{v})|^2\right| \leq \frac{|\Psi(\mathbf{v})|^2}{\mathrm{poly}(n)} + \frac{c}{2^{mn}} \tag{4}$$

where $0 \leq c < 1/2$ and the lattice size is $n \times m$. This equation implies computing $|\widetilde{\Psi}(\mathbf{v})|^2$ is also #P-hard such that

$$\left||\Psi(\mathbf{v})|^2 - |\widetilde{\Psi}(\mathbf{v})|^2\right| \leq \frac{c}{2^{mn}}. \tag{5}$$

Denote $\mathcal{O}$ as an oracle with the ability to compute the first $mn-1$ digits of $\Psi(\mathbf{v})$. The above statement implies

$$\mathsf{P}^{\#P} \subseteq \mathsf{P}^{\mathcal{O}}. \tag{6}$$

If $|\psi_{\mathrm{GWD}}\rangle$ can be represented efficiently by a RBM, calculating its wave function in the computational basis belongs to the complexity class $\mathsf{P/poly}$ as discussed in the main text. Thus $\mathcal{O} \subseteq \mathsf{P/poly}$. Combining these results, we have

Lemma 1 RBM cannot represent $|\psi_{\mathrm{GWD}}\rangle$ exactly unless

$$\mathsf{P}^{\#P} \subseteq \mathsf{P}^{\mathsf{P/poly}}. \tag{7}$$

Supplementary Ref. [8] proves if the containment in the above equation is true, the polynomial hierarchy will collapse [5], which is widely believed to be unlikely.

Approximate representation

Denote a measurement in the computational basis as a quantum operator $\mathcal{E}$ and $\widetilde{\Psi}(\mathbf{v})$ as the wave function of a RBM state $|\psi'_{\mathrm{GWD}}\rangle$ to approximate $|\psi_{\mathrm{GWD}}\rangle$, then we have

$$\sum_{\mathbf{v}} \left| |\Psi(\mathbf{v})|^2 - |\widetilde{\Psi}(\mathbf{v})|^2 \right| = 2D(\mathcal{E}(|\psi_{\mathrm{GWD}}\rangle\langle\psi_{\mathrm{GWD}}|), \mathcal{E}(|\psi'_{\mathrm{GWD}}\rangle\langle\psi'_{\mathrm{GWD}}|)) \leq 2D(|\psi_{\mathrm{GWD}}\rangle\langle\psi_{\mathrm{GWD}}|, |\psi'_{\mathrm{GWD}}\rangle\langle\psi'_{\mathrm{GWD}}|) \leq \epsilon, \quad (8)$$

where $D(\rho_1, \rho_2)$ denotes the trace distance, defined as $\mathrm{tr}|\rho_1 - \rho_2|/2$. The above equation means if the trace distance between $|\psi_{\mathrm{GWD}}\rangle$ and $|\psi'_{\mathrm{GWD}}\rangle$ is smaller than $\epsilon/2$, we have $\mathbb{E}_{\mathbf{v}}\left[\left| |\Psi(\mathbf{v})|^2 - |\widetilde{\Psi}(\mathbf{v})|^2 \right|\right] \leq \epsilon/2^{mn}$ where $\mathbb{E}_{\mathbf{v}}[f(\mathbf{v})]$ means the expectation value of $f(\mathbf{v})$ over uniform distribution of $\mathbf{v}$. Using the Markov inequality, we get

$$\Pr_{\mathbf{v}}\left[\left| |\Psi(\mathbf{v})|^2 - |\widetilde{\Psi}(\mathbf{v})|^2 \right| \geq \frac{\epsilon}{2^{mn}\delta}\right] \leq \delta, \quad (9)$$

where $\epsilon/\delta < 1/2$ and $\Pr_{\mathbf{v}}[f(\mathbf{v})]$ denotes the probability such that $\mathbf{v}$ satisfies condition $f(\mathbf{v})$ if random variable $\mathbf{v}$ is uniform distributed. This equation means that for $1-\delta$ fraction of the whole set $\mathbf{v}$, we have

$$\left| |\Psi(\mathbf{v})|^2 - |\widetilde{\Psi}(\mathbf{v})|^2 \right| \leq \frac{\epsilon}{2^{mn}\delta}. \quad (10)$$

Denote $\mathcal{O}'$ as an oracle with the ability to compute the first $mn-1$ digits of $|\Psi(\mathbf{v})|^2$ for such fraction of $\mathbf{v}$, then $\mathcal{O}' \subseteq \mathcal{O} \subseteq \mathsf{P/poly}$.

In Supplementary Ref. [6], we introduced a conjecture that #P-hardness of approximating $\Psi(\mathbf{v})$ to the error given by Supplementary Eq. (10) still holds when we lift from the worst-case to the average-case, that is,

Conjecture 1 For any $1-\delta$ fraction of instance $\mathbf{v}$, approximating $|\Psi(\mathbf{v})|^2$ by $|\widetilde{\Psi}(\mathbf{v})|^2$ up to the error

$$\left| |\Psi(\mathbf{v})|^2 - |\widetilde{\Psi}(\mathbf{v})|^2 \right| \leq \frac{\epsilon}{2^{mn}\delta}$$

is still #P-hard.

Supplementary Ref. [6] has discussed why this is a reasonable conjecture. It is related to classical-hardness for simulating distribution from random quantum circuit and is supported by both quantum chaos theory and extensive numerical simulations [9]. With this conjecture, we have

$$\mathsf{P}^{\#P} \subseteq \mathsf{P}^{\mathcal{O}'}. \quad (11)$$

Combining the results above, we have the following theorem

Lemma 2 If the conjecture 1 is true, any RBM states cannot approximate $|\psi_{\mathrm{GWD}}\rangle$ with the trace distance smaller than $\epsilon/2$, otherwise $\mathsf{P}^{\#P} \subseteq \mathsf{P}^{\mathsf{P/poly}}$ and the polynomial hierarchy collapses.

Supplementary Note 4

A comment on exact and approximate representations of local tensors

In the proof of Theorem 3 in the main text, we use the universal quantum computing gadget to construct an exact neural network representation of local tensors in the tensor network state. We can also use the universal approximation theorem [10] to construct an approximate representation of local tensors as in Supplementary Ref.[11]. However, we should note that for the tensor network state, some property of the state is extremely sensitive to the approximation in the local tensors. For instance, for the toric code state, we have the tensor network representation with the exact local tensors given by

$$\begin{aligned} g(v_1, v_2, v_3, v_4) &= 1 \text{ if } v_1 + v_2 + v_3 + v_4 \text{ is even}, \\ g(v_1, v_2, v_3, v_4) &= 0 \text{ if } v_1 + v_2 + v_3 + v_4 \text{ is odd}. \end{aligned} \quad (12)$$

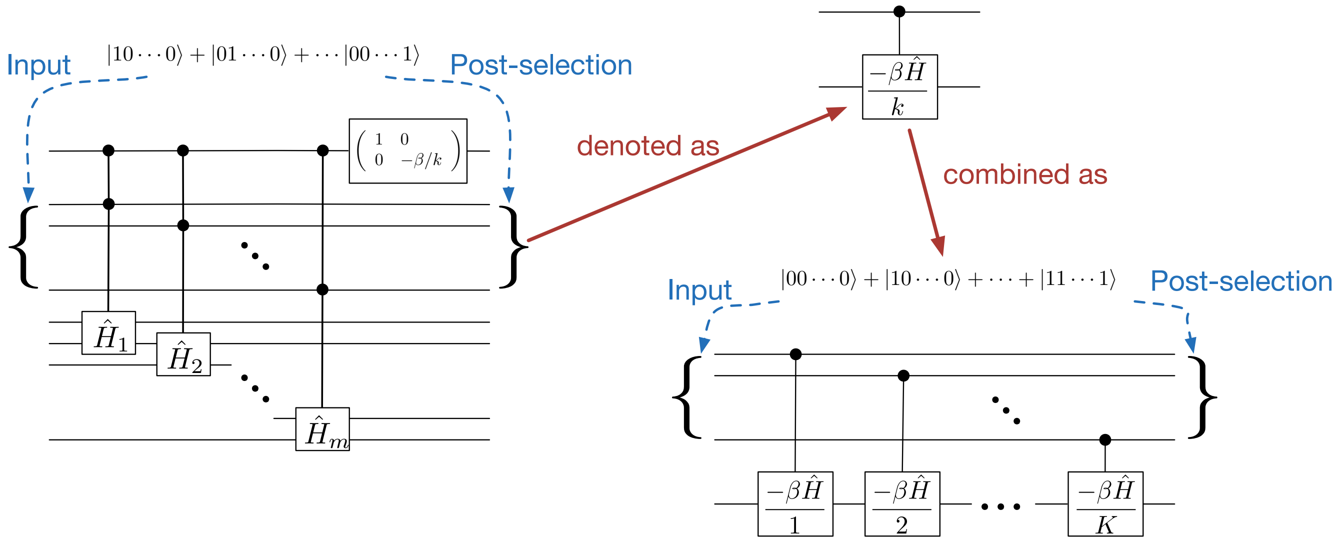

Supplementary Fig. 3: **Construction of a pseudo quantum circuit for DBM representation of ground states of many-body Hamiltonians.** We Construct a pseudo quantum circuit where each elementary gate represents a linear but non-unitary operation which can be described through multiplication of a local tensor. The left diagram represents construction of the pseudo-gate controlled-$(-\beta\hat{H}/k)$, which acts as a basic building block for construction of the pseudo-gate $e^{-\beta\hat{H}}$ shown on the lower right side through the Taylor series expansion.

If we approximate the local tensors by the following expression

$$\begin{aligned} g(v_1, v_2, v_3, v_4) &= 1 \text{ if } v_1 + v_2 + v_3 + v_4 \text{ is even}, \\ g(v_1, v_2, v_3, v_4) &= \epsilon \text{ if } v_1 + v_2 + v_3 + v_4 \text{ is odd}. \end{aligned} \tag{13}$$

where ϵ can be an arbitrarily small constant, it is shown In Supplementary Ref. [12] that as long as $\epsilon \neq 0$, the topological entanglement entropy vanishes in the thermodynamic limit. So the existence of topological entanglement entropy is extremely sensitive to approximation in the local tensors for this tensor network state. The reason for this is that the approximation error in each local tensor is amplified when we connect the local tensors to build up the global tensor network state, in particular when we go to the thermodynamical limit where the number of connected local tensors goes to infinity. So we prefer the use of exact representation of the local tensors as we did in theorem 3. If one uses the approximate representation, one should bound the total error in the resultant tensor network state (instead of the error of each individual local tensor) to be small.

Supplementary Note 5

Efficient tensor network representation for ground states

In this section, we prove the theorem 4 in the main text. For this purpose, we develop a method using Taylor series expansion to efficiently construct ground state of any Hamiltonian with tensor network (and thus DBM network as well due to the theorem 3). Compared with the previous construction method [13], this approach allows an exponential improvement on the scaling with respect to the precision of the representation. We use pseudo quantum circuit to present our construction as shown in Supplementary Fig. 3. Pseudo quantum circuit is similar to conventional quantum circuit except that the pseudo-gate is not required to be unitary. Each pseudo-gate still represents a linear transformation through matrix multiplication, so it can be easily constructed through a local tensor. The pseudo quantum circuit then just represents a tensor network.

Suppose the number of interaction terms in the Hamiltonian is m, i.e., $\hat{H} \equiv \sum_{i=1}^{m} \hat{H}_i$, where each $\hat{H}_i$ involves at most k-body interactions (k is typically a small constant. For instance, $k = 2$ for any Hamiltonian with two-body

interaction. The interaction can be long-range.) We simulate the operator $\hat{H}$ by first generating a state $\sum_{i=1}^{m}|i\rangle$, where $|i\rangle \equiv |0_1 0_2 \cdots 1_i \cdots 0_m\rangle$, i.e., only the i-th bit is 1. Applying the operation $\hat{H}_i$ controlled by the i-th qubit as shown in Supplementary Fig. 3 and post-selecting the control bits in the state $\sum_{i=1}^{m}|i\rangle$ (note that postselection can be easily represented in a tensor network), we get

$$\sum_{i=1}^{m}|i\rangle \xrightarrow{\text{controlled-}\hat{H}_i} \sum_{i=1}^{m}|i\rangle\hat{H}_i \xrightarrow{\text{post-selection}} \sum_{i=1}^{m}\hat{H}_i. \tag{14}$$

This requires $O(m)$ pseudo quantum gates as shown in Supplementary Fig. 3. All of the above operations are controlled by an additional qubit (see Supplementary Fig. 3). We then apply a non-unitary matrix $\text{diag}(1, -\beta/k)$ on this control qubit, and construct a pseudo-gate controlled-$(-\beta\hat{H}/k)$. Note that this gate requires $O(m)$ elementary pseudo quantum gates for its construction and we use the pseudo-gate controlled-$(-\beta\hat{H}/k)$ as a building block for the next step. In the next step, we first generate a superposition state $\sum_{k=0}^{K}|k\rangle$, where K corresponds to the truncation number in Taylor series expansion and $|k\rangle \equiv |1_1 1_2 \cdots 1_k 0_{k+1} \cdots 0_K\rangle$, i.e. only the first k bits are in state $|1\rangle$. Applying the circuit shown in Supplementary Fig. 3 and post-selecting the output state of the control qubits in $\sum_{k=0}^{K}|k\rangle$, we get

$$\sum_{k=0}^{K}|k\rangle \xrightarrow{\text{controlled-}(-\beta\hat{H}/k)} \sum_{k=0}^{K}|k\rangle\frac{(-\beta\hat{H})^k}{k!} \xrightarrow{\text{post-selection}} \sum_{k=0}^{K}\frac{(-\beta\hat{H})^k}{k!} \tag{15}$$

which is the Taylor expansion of $e^{-\beta\hat{H}}$ truncated to the K-th order. Note that this construction of $e^{-\beta\hat{H}}$ requires $O(Km)$ elementary pseudo quantum gates.

We apply the above operator $e^{-\beta\hat{H}}$ on the state

$$\sum_{i=0}^{2^n-1}|i\rangle|i\rangle = \sum_{i=0}^{2^n-1}|\psi_i\rangle|\psi_i^*\rangle \tag{16}$$

where n represents the total number of qubits in the Hamiltonian $\hat{H}$, $|\psi_i\rangle$ denotes the i-th eigenstate of $\hat{H}$, and $|\psi_i^*\rangle$ is the complex conjugate of $|\psi_i\rangle$ in the computational basis. After tracing out the register storing $|\psi_i^*\rangle$ and dropping the unimportant normalization factor, we get the state

$$|\psi_0\rangle\langle\psi_0| + O\left(2^n e^{-\beta\Delta} + \frac{2^n(\beta\|\hat{H}\|)^K}{K!}\right) \tag{17}$$

for the first register. In the equation above, the first term represents our targeted ground state $|\psi_0\rangle$, and there are two error terms: the first term comes from contribution of all the other eigenstates, shrunk by the imaginary time evolution factor $e^{-\beta\Delta}$ with Δ denoting the energy gap; and the second term comes from the truncation error in Taylor series expansion. Suppose we require the total representation error is bounded by a small constant ϵ, then we need

$$O\left(2^n e^{-\beta\Delta}\right) \leq \frac{\epsilon}{2}, \tag{18}$$

$$O\left(\frac{2^n(\beta\|\hat{H}\|)^K}{K!}\right) \leq \frac{\epsilon}{2}. \tag{19}$$

With an optimal choice of the parameter $\beta = O((n + \log(1/\epsilon))/\Delta)$, we need $K = O(\beta\|\hat{H}\|)$ to satisfy these inequalities. As $\|\hat{H}\| = O(m)$, we have $K = O((n + \log(1/\epsilon))m/\Delta)$. So the total number of elementary tensors we need to represent the ground state $|\psi_0\rangle$ of the Hamiltonian $\hat{H}$ is given by $O(Km)$, which is

$$O\left(\frac{1}{\Delta}\left(n + \log\frac{1}{\epsilon}\right)m^2\right). \tag{20}$$

Each elementary tensor has a constant bond dimension D and a typically small coordination number d. Actually, we can choose $D = 2$ and $d = 4$ because each interaction term in the Hamiltonian can be expressed as tensor product of k Pauli matrices and thus has a unitary form. The elementary tensor then has the controlled-controlled-unitary form, which can be decomposed into a small sequence of two-qubit unitary gates [14, 15]. Each two-qubit unitary gate can be expressed as a tensor with $D = 2$ and $d = 4$. Combining with theorem 3 in the main text, we find the total number of neurons in the DBM to represent the ground state of the Hamiltonian $\hat{H}$ is also given by the above equation, which is the statement of theorem 4.

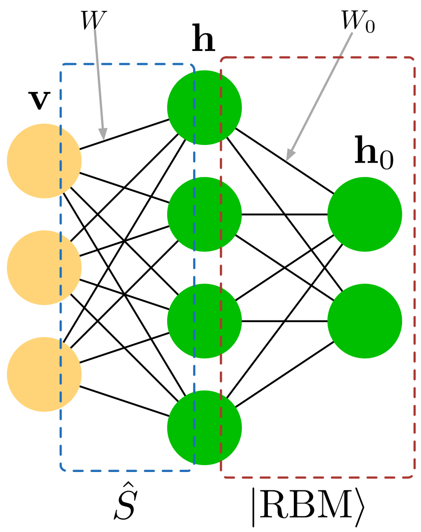

Supplementary Fig. 4: **Connection between DBM and RBM representations.** In this notation for the DBM representation, $\mathbf{v}, \mathbf{h}, \mathbf{h}_0$ denote the values of visible neurons, neurons of first hidden layers, neurons of second hidden layers, respectively, and $\mathbf{W}, \mathbf{W}_0$ are correlation matrices between successive layers in the weight function. In the Monte Carlo sampling algorithm, we sample the variables $\mathbf{h}$. In this case, we regard the combination of two hidden layers as a RBM with visible neurons $\mathbf{h}$ and hidden neurons $\mathbf{h}_0$. Operator $\hat{S}$ is used to map the RBM into the DBM of which matrix elements in the basis $\mathbf{v}$ and $\mathbf{h}$ are given by the exponent of $\mathbf{v}^T \mathbf{W} \mathbf{h}$.

Supplementary Note 6

A Prototype Algorithm for Training DBM

Our constructive proof of theorems 2 and 4 gives a systematic approach to construct an efficient (with a polynomial number of neurons or parameters) DBM representation for any states from quantum dynamics or ground states of physical Hamiltonians. However, they don't address the question on how to extract properties of physical observables from the DBM representation. Furthermore, significantly more succinct neural network representation (either by RBM or by DBM) could exist for practical quantum states than what indicated by those general theorems. To address this question, in this section we give a prototype algorithm for training DBM by the gradient descent (reinforcement learning) method to directly approximate ground states of given Hamiltonians. This algorithm is similar to the algorithm proposed recently in Supplementary Ref. [16] for training RBM: it is also based on Monte Carlo sampling such that computing variational derivative can be reduced to computing expectation values of some easily computable function over a probability distribution. The probability distribution can be sampled with a Markov chain, and for each sample from this probability distribution, the function (corresponding to the expectation value of some physical observable) can be computed efficiently.

We emphasize that this algorithm is only considered to be a prototype as it has not been numerically tested and could be significantly improved for real problems. The algorithm is not guaranteed to be efficient. From the computational complexity theory, the general contraction problem for either RBM or DBM (or tensor network) representation is at least NP-hard [17], so we don't expect there is always an efficient algorithm to extract physics observables in the worst case. The general NP-hardness about the contraction of course does not prevent the use of RBM or DBM learning algorithms for solving real problems as the typical-case hardness could be very different from the worst-case hardness. Supplementary Ref. [16] has shown the learning algorithm based on RBM has successfully solved several spin models. By borrowing ideas and numerical techniques from the deep learning and other machine learning literature for solving practical problems [18, 19], we expect that the learning algorithms based on RBM or DBM representation of quantum states will attract a lot of interest in the coming years.

Notations

The wave function of the quantum state represented by a DBM can be written as

$$\langle \mathbf{v}|\mathrm{DBM}\rangle = \sum_{\mathbf{h}} e^{\mathbf{v}^T\mathbf{W}\mathbf{h}} \sum_{\mathbf{h}_0} e^{\mathbf{h}^T\mathbf{W}_0\mathbf{h}_0} \tag{21}$$

as shown in Supplementary Fig. 4, where $\mathbf{v}, \mathbf{h}, \mathbf{h}_0$ are the values of visible neurons, neurons of first hidden layers, neurons of second hidden layers respectively and $\mathbf{W}, \mathbf{W}_0$ are correlation matrices between successive layers in weight function. Without loss of generality and for simplification of the notation, we omit the bias term (linear term in the exponent) here. Notice that the last summation term can be regarded as wave function of a RBM with visible neurons $\mathbf{h}$ and hidden neurons $\mathbf{h}_0$. For convenience of notation, we denote a DBM state as

$$|\mathrm{DBM}\rangle = \hat{S}|\mathrm{RBM}\rangle \tag{22}$$

where

$$\langle \mathbf{v}|\hat{S}|\mathbf{h}\rangle = e^{\mathbf{v}^T\mathbf{W}\mathbf{h}} \tag{23}$$

$$\langle \mathbf{h}|\mathrm{RBM}\rangle = \sum_{\mathbf{h}_0} e^{\mathbf{h}^T\mathbf{W}_0\mathbf{h}_0}. \tag{24}$$

Where the symbol $|\mathrm{RBM}\rangle$ is only used to simplify the notation and does not correspond to a real quantum state represented by a RBM.

Extracting physical observables

Here, we give a Monte Carlo algorithm to compute expectation value of a physical observable O from the DBM state.

$$\begin{aligned}\langle O\rangle &= \frac{\langle \mathrm{DBM}|O|\mathrm{DBM}\rangle}{\langle \mathrm{DBM}|\mathrm{DBM}\rangle} \\ &= \frac{\langle \mathrm{RBM}|\hat{S}^\dagger O\hat{S}|\mathrm{RBM}\rangle}{\langle \mathrm{RBM}|\hat{S}^\dagger\hat{S}|\mathrm{RBM}\rangle} \\ &= \frac{\langle \mathrm{RBM}|\hat{S}^\dagger O\hat{S}|\mathrm{RBM}\rangle/\langle \mathrm{RBM}|\mathrm{RBM}\rangle^2}{\langle \mathrm{RBM}|\hat{S}^\dagger\hat{S}|\mathrm{RBM}\rangle/\langle \mathrm{RBM}|\mathrm{RBM}\rangle^2}\end{aligned} \tag{25}$$

We consider the numerator and the denominator separately.

For the numerator,

$$\begin{aligned}\frac{\langle \mathrm{RBM}|\hat{S}^\dagger O\hat{S}|\mathrm{RBM}\rangle}{\langle \mathrm{RBM}|\mathrm{RBM}\rangle^2} &= \sum_{\mathbf{h},\mathbf{h}'} \frac{\langle \mathrm{RBM}|\mathbf{h}\rangle\langle \mathbf{h}|\hat{S}^\dagger O\hat{S}|\mathbf{h}'\rangle\langle \mathbf{h}'|\mathrm{RBM}\rangle}{\langle \mathrm{RBM}|\mathrm{RBM}\rangle^2} \\ &= \sum_{\mathbf{h},\mathbf{h}'} \frac{|\langle \mathrm{RBM}|\mathbf{h}\rangle|^2}{\langle \mathrm{RBM}|\mathrm{RBM}\rangle}\frac{|\langle \mathrm{RBM}|\mathbf{h}'\rangle|^2}{\langle \mathrm{RBM}|\mathrm{RBM}\rangle}\frac{\langle \mathbf{h}|\hat{S}^\dagger O\hat{S}|\mathbf{h}'\rangle}{\langle \mathbf{h}|\mathrm{RBM}\rangle\langle \mathrm{RBM}|\mathbf{h}'\rangle} \\ &= \sum_{\mathbf{h},\mathbf{h}'} p_{\mathbf{h}}p_{\mathbf{h}'}\mathcal{O}_{\mathbf{h},\mathbf{h}'}\end{aligned} \tag{26}$$

where

$$p_{\mathbf{h}} = \frac{|\langle \mathrm{RBM}|\mathbf{h}\rangle|^2}{\langle \mathrm{RBM}|\mathrm{RBM}\rangle}, \quad p_{\mathbf{h}'} = \frac{|\langle \mathrm{RBM}|\mathbf{h}'\rangle|^2}{\langle \mathrm{RBM}|\mathrm{RBM}\rangle}, \quad \mathcal{O}_{\mathbf{h},\mathbf{h}'} = \frac{\langle \mathbf{h}|\hat{S}^\dagger O\hat{S}|\mathbf{h}'\rangle}{\langle \mathbf{h}|\mathrm{RBM}\rangle\langle \mathrm{RBM}|\mathbf{h}'\rangle} \tag{27}$$

Since wave function of RBM can be factorized given a specific configuration (a specific value assignment to neurons $\mathbf{h}$ and $\mathbf{h}'$), the ratio $p_{\mathbf{h}}p_{\mathbf{h}'}/p_{\mathbf{h}_1}p_{\mathbf{h}'_1}$ ($\mathbf{h}_1, \mathbf{h}'_1$ denotes a new configuration) is easy to compute. Then we can use Metropolis algorithm to sample the distribution:

$$\Pr(\mathbf{h}\mathbf{h}' \to \mathbf{h}_1\mathbf{h}'_1) = \min\left(1, \frac{p_{\mathbf{h}_1}p_{\mathbf{h}'_1}}{p_{\mathbf{h}}p_{\mathbf{h}'}}\right) \tag{28}$$

where $\Pr(\mathbf{h}\mathbf{h}' \to \mathbf{h}_1\mathbf{h}_1')$ denotes the probability to change the configuration from $\mathbf{h}\mathbf{h}'$ to $\mathbf{h}_1\mathbf{h}_1'$.

The function $\mathcal{O}_{\mathbf{h},\mathbf{h}'}$ can be computed efficiently given the configuration $\mathbf{h}$ and $\mathbf{h}'$. Suppose O is decomposed as a tensor product of at most k Pauli matrices, where k is typically a small constant. Denote $|\bar{\mathbf{v}}\rangle$ as computational basis of Hilbert space acted trivially by O and the remaining as $|\mathbf{v}_0\rangle, |\mathbf{v}_0'\rangle \in \{0,1\}^k$. Thus

$$\langle \mathbf{v}|\hat{S}|\mathbf{h}\rangle = \langle \mathbf{v}_0, \bar{\mathbf{v}}|\hat{S}|\mathbf{h}\rangle = e^{(\mathbf{v}_0^T, \bar{\mathbf{v}}^T)\mathbf{W}\mathbf{h}} = e^{\mathbf{v}_0^T\mathbf{W}_1\mathbf{h} + \bar{\mathbf{v}}^T\mathbf{W}_2\mathbf{h}} = \langle \bar{\mathbf{v}}|\hat{\bar{S}}|\mathbf{h}\rangle\langle \mathbf{v}_0|\hat{S}_0|\mathbf{h}\rangle \tag{29}$$

where

$$\begin{aligned} \langle \mathbf{v}_0|\hat{S}_0|\mathbf{h}\rangle &= e^{\mathbf{v}_0^T\mathbf{W}_1\mathbf{h}} && (30)\\ \langle \bar{\mathbf{v}}|\hat{\bar{S}}|\mathbf{h}\rangle &= e^{\bar{\mathbf{v}}^T\mathbf{W}_2\mathbf{h}}. && (31)\end{aligned}$$

Then we have

$$\begin{aligned} \mathcal{O}_{\mathbf{h},\mathbf{h}'} &= \frac{\langle \mathbf{h}|\hat{S}^\dagger O\hat{S}|\mathbf{h}'\rangle}{\langle \mathbf{h}|\mathrm{RBM}\rangle\langle \mathrm{RBM}|\mathbf{h}'\rangle}\\ &= \frac{\sum_{\mathbf{v},\mathbf{v}'}\langle \mathbf{h}|\hat{S}^\dagger|\mathbf{v}\rangle\langle \mathbf{v}|O|\mathbf{v}'\rangle\langle \mathbf{s}'|\hat{S}|\mathbf{h}'\rangle}{\langle \mathbf{h}|\mathrm{RBM}\rangle\langle \mathrm{RBM}|\mathbf{h}'\rangle}\\ &= \frac{\sum_{\mathbf{v}_0,\mathbf{v}_0'}\langle \mathbf{h}|\hat{S}_0^\dagger|\mathbf{v}_0\rangle\langle \mathbf{v}_0|O|\mathbf{v}_0'\rangle\langle \mathbf{v}_0'|\hat{S}_0|\mathbf{h}'\rangle\sum_{\bar{\mathbf{v}}}\langle \mathbf{h}|\hat{\bar{S}}^\dagger|\bar{\mathbf{v}}\rangle\langle \bar{\mathbf{v}}|\hat{\bar{S}}|\mathbf{h}'\rangle}{\langle \mathbf{h}|\mathrm{RBM}\rangle\langle \mathrm{RBM}|\mathbf{h}'\rangle} && (32)\end{aligned}$$

The first summation in the numerator only contains $2^{2k} = O(1)$ terms. The second one is basically wave function of RBM with hidden neurons $\bar{\mathbf{v}}$ and visible neurons $(\mathbf{h}, \mathbf{h}')$ because

$$\sum_{\bar{\mathbf{v}}}\langle \mathbf{h}|\hat{\bar{S}}^\dagger|\bar{\mathbf{v}}\rangle\langle \bar{\mathbf{v}}|\hat{\bar{S}}|\mathbf{h}'\rangle = \sum_{\bar{\mathbf{v}}} e^{\bar{\mathbf{v}}^T\mathbf{W}_2^*\mathbf{h} + \bar{\mathbf{v}}^T\mathbf{W}_2\mathbf{h}'}. \tag{33}$$

Thus, function $\mathcal{O}_{\mathbf{h},\mathbf{h}'}$ can be computed from wave functions of three RBMs.

The denominator of Supplementary Eq.(25) has the same form as the numerator

$$\frac{\langle \mathrm{RBM}|\hat{S}^\dagger\hat{S}|\mathrm{RBM}\rangle}{\langle \mathrm{RBM}|\mathrm{RBM}\rangle} = \sum_{\mathbf{h},\mathbf{h}'} p_{\mathbf{h}}p_{\mathbf{h}'}\mathcal{I}_{\mathbf{h},\mathbf{h}'}, \tag{34}$$

except for the last term

$$\mathcal{I}_{\mathbf{h},\mathbf{h}'} = \frac{\langle \mathbf{h}|\hat{S}^\dagger\hat{S}|\mathbf{h}'\rangle}{\langle \mathbf{h}|\mathrm{RBM}\rangle\langle \mathrm{RBM}|\mathbf{h}'\rangle} \tag{35}$$

which, similar to $\mathcal{O}_{\mathbf{h},\mathbf{h}'}$, can also be computed from multiplication of wave functions of three RBMs.

Energy minimization through the gradient decent method

The energy of the system is given by the expectation value of the Hamiltonian H

$$\langle H\rangle = \frac{\langle \mathrm{RBM}|\hat{S}^\dagger H\hat{S}|\mathrm{RBM}\rangle/\langle \mathrm{RBM}|\mathrm{RBM}\rangle^2}{\langle \mathrm{RBM}|\hat{S}^\dagger\hat{S}|\mathrm{RBM}\rangle/\langle \mathrm{RBM}|\mathrm{RBM}\rangle^2} = \frac{A}{B} \tag{36}$$

where

$$\begin{aligned} A &= \frac{\langle \mathrm{RBM}|\hat{S}^\dagger H\hat{S}|\mathrm{RBM}\rangle}{\langle \mathrm{RBM}|\mathrm{RBM}\rangle^2} && (37)\\ B &= \frac{\langle \mathrm{RBM}|\hat{S}^\dagger\hat{S}|\mathrm{RBM}\rangle}{\langle \mathrm{RBM}|\mathrm{RBM}\rangle^2} && (38)\end{aligned}$$

To minimize the energy, we minimize the numerator A while keeping B almost constant. We take the gradient decent method for which the change of parameters $\eta\mathbf{w}$ should be along the direction

$$\max_{\mathbf{w}} -\partial A \cdot \mathbf{w} \tag{39}$$
$$\partial B \cdot \mathbf{w} = 0 \tag{40}$$
$$\mathbf{w} \cdot \mathbf{w} = 1 \tag{41}$$

where η denotes a small step size or learning rate and $\mathbf{w}$ denotes the direction of parameter changes. The optimal direction $\mathbf{w}$ is easy to calculate if we know the variational derivatives ∂A and ∂B.

First, we consider the derivative ∂A

$$\begin{aligned} \partial A &= \partial \frac{\langle \mathrm{RBM}|\hat{S}^\dagger H \hat{S}|\mathrm{RBM}\rangle}{\langle \mathrm{RBM}|\mathrm{RBM}\rangle^2} \\ &= \frac{\partial\langle \mathrm{RBM}|\hat{S}^\dagger H \hat{S}|\mathrm{RBM}\rangle}{\langle \mathrm{RBM}|\mathrm{RBM}\rangle^2} - \frac{2\langle \mathrm{RBM}|\hat{S}^\dagger H \hat{S}|\mathrm{RBM}\rangle \partial \langle \mathrm{RBM}|\mathrm{RBM}\rangle}{\langle \mathrm{RBM}|\mathrm{RBM}\rangle^3}. \end{aligned} \tag{42}$$

We consider the change of $|\mathrm{RBM}\rangle$ (represented by the weight function $\mathbf{W}_0$) and $\hat{S}$ (represented by the weight function $\mathbf{W}$) separately:

$$\begin{aligned} \text{change of } \hat{S} : \partial_{\mathbf{W}_0} A &= \sum_{\mathbf{h},\mathbf{h}'} \frac{\langle \mathrm{RBM}|\mathbf{h}\rangle\langle \mathbf{h}'|\mathrm{RBM}\rangle \partial_{\mathbf{W}_0}\langle \mathbf{h}|\hat{S}^\dagger H \hat{S}|\mathbf{h}'\rangle}{\langle \mathrm{RBM}|\mathrm{RBM}\rangle^2} \\ &= \sum_{\mathbf{h},\mathbf{h}'} p_{\mathbf{h}} p_{\mathbf{h}'} \partial \mathcal{H}_{\mathbf{h},\mathbf{h}'} \end{aligned} \tag{43}$$

where

$$\partial \mathcal{H}_{\mathbf{h},\mathbf{h}'} = \frac{\partial_{\mathbf{W}_0}\langle \mathbf{h}|\hat{S}^\dagger H \hat{S}|\mathbf{h}'\rangle}{\langle \mathbf{h}|\mathrm{RBM}\rangle\langle \mathrm{RBM}|\mathbf{h}'\rangle} \tag{44}$$

can be computed efficiently since the numerator is derivative of a function which has closed algebraic form of polynomial size. Note that $\langle \mathbf{h}|\hat{S}^\dagger H \hat{S}|\mathbf{h}'\rangle = \sum_i^m \langle \mathbf{h}|\hat{S}^\dagger H_i \hat{S}|\mathbf{h}'\rangle$ where H_i is local observable and each term has the same form as Supplementary Eq.(32), which is efficiently computable.

Similarly,

$$\begin{aligned} \text{change of } |\mathrm{RBM}\rangle : \partial_{\mathbf{W}} A &= \sum_{\mathbf{h},\mathbf{h}'} \frac{(\partial_{\mathbf{W}}\langle \mathrm{RBM}|\mathbf{h}\rangle)\langle \mathbf{h}|\hat{S}^\dagger H \hat{S}|\mathbf{h}'\rangle\langle \mathbf{h}'|\mathrm{RBM}\rangle}{\langle \mathrm{RBM}|\mathrm{RBM}\rangle^2} + \\ &\sum_{\mathbf{h},\mathbf{h}'} \frac{\langle \mathrm{RBM}|\mathbf{h}'\rangle\langle \mathbf{h}'|\hat{S}^\dagger H \hat{S}|\mathbf{h}\rangle(\partial_{\mathbf{W}}\langle \mathbf{h}|\mathrm{RBM}\rangle)}{\langle \mathrm{RBM}|\mathrm{RBM}\rangle^2} - \\ &\sum_{\mathbf{h},\mathbf{h}'} \frac{\langle \mathrm{RBM}|\mathbf{h}\rangle\langle \mathbf{h}|\hat{S}^\dagger H \hat{S}|\mathbf{h}'\rangle\langle \mathbf{h}'|\mathrm{RBM}\rangle}{\langle \mathrm{RBM}|\mathrm{RBM}\rangle^2} \sum_{\mathbf{h}''} \frac{(\partial_{\mathbf{W}}\langle \mathrm{RBM}|\mathbf{h}''\rangle)\langle \mathbf{h}''|\mathrm{RBM}\rangle}{\langle \mathrm{RBM}|\mathrm{RBM}\rangle} - \\ &\sum_{\mathbf{h},\mathbf{h}'} \frac{\langle \mathrm{RBM}|\mathbf{h}\rangle\langle \mathbf{h}|\hat{S}^\dagger H \hat{S}|\mathbf{h}'\rangle\langle \mathbf{h}'|\mathrm{RBM}\rangle}{\langle \mathrm{RBM}|\mathrm{RBM}\rangle^2} \sum_{\mathbf{h}''} \frac{\langle \mathrm{RBM}|\mathbf{h}''\rangle(\partial_{\mathbf{W}}\langle \mathbf{h}''|\mathrm{RBM}\rangle)}{\langle \mathrm{RBM}|\mathrm{RBM}\rangle} \end{aligned} \tag{45}$$

$$= \sum_{\mathbf{h},\mathbf{h}'} p_{\mathbf{h}} p_{\mathbf{h}'} \left(\beta_{\mathbf{h}} \mathcal{H}_{\mathbf{h},\mathbf{h}'} + \beta_{\mathbf{h}}^* \mathcal{H}_{\mathbf{h},\mathbf{h}'}^*\right) - \sum_{\mathbf{h},\mathbf{h}'} p_{\mathbf{h}} p_{\mathbf{h}'} \mathcal{H}_{\mathbf{h},\mathbf{h}'} \sum_{\mathbf{h}''} p_{\mathbf{h}''}(\beta_{\mathbf{h}''} + \beta_{\mathbf{h}''}^*) \tag{46}$$

where

$$\beta_{\mathbf{h}} = \frac{\partial_{\mathbf{W}}\langle \mathrm{RBM}|\mathbf{h}\rangle}{\langle \mathrm{RBM}|\mathbf{h}\rangle}, \quad \mathcal{H}_{\mathbf{h},\mathbf{h}'} = \frac{\langle \mathbf{h}|\hat{S}^\dagger H \hat{S}|\mathbf{h}'\rangle}{\langle \mathbf{h}|\mathrm{RBM}\rangle\langle \mathrm{RBM}|\mathbf{h}'\rangle} \tag{47}$$

where $\beta_{\mathbf{h}}$ can be computed efficiently since its numerator is derivative of the wave function of a RBM.

Using similar decomposition, we can express the derivative ∂B as

$$\partial_{\mathbf{W}_0} B = \sum_{\mathbf{h},\mathbf{h}'} p_{\mathbf{h}} p_{\mathbf{h}'} \partial \mathcal{I}_{\mathbf{h},\mathbf{h}'} \tag{48}$$

$$\partial_{\mathbf{W}} B = \sum_{\mathbf{h},\mathbf{h}'} p_{\mathbf{h}} p_{\mathbf{h}'} \left(\beta_{\mathbf{h}} \mathcal{I}_{\mathbf{h},\mathbf{h}'} + \beta^*_{\mathbf{h}} \mathcal{I}^*_{\mathbf{h},\mathbf{h}'}\right) - \sum_{\mathbf{h},\mathbf{h}'} p_{\mathbf{h}} p_{\mathbf{h}'} \mathcal{I}_{\mathbf{h},\mathbf{h}'} \sum_{\mathbf{h}''} p_{\mathbf{h}''} (\beta_{\mathbf{h}''} + \beta^*_{\mathbf{h}''}) \tag{49}$$

where

$$\partial \mathcal{I}_{\mathbf{h},\mathbf{h}'} = \frac{\partial_{\mathbf{W}_0} \langle \mathbf{h} | \hat{S}^\dagger \hat{S} | \mathbf{h}' \rangle}{\langle \mathbf{h} | \mathrm{RBM} \rangle \langle \mathrm{RBM} | \mathbf{h}' \rangle}. \tag{50}$$

Given a configuration $\mathbf{h}, \mathbf{h}'$ of the probability distribution, the functions $\partial \mathcal{I}_{\mathbf{h},\mathbf{h}'}$ et al. can be computed efficiently, and the probability distribution $p_{\mathbf{h}} p_{\mathbf{h}'}$ can be sampled with the Metropolis algorithm as we deailed before. So this gives a Monte Carlo sampling method to calcuate the devative of the energy, which, combined with the gradient decent method, provides a way to directly learn the DBM wave function for the ground state of the Hamiltonian.

Supplementary References

[1] Vitagliano, G., Riera, A. & Latorre, J. Volume-law scaling for the entanglement entropy in spin-1/2 chains. *New Journal of Physics* **12**, 113049 (2010).

[2] Verstraete, F., Wolf, M. M., Perez-Garcia, D. & Cirac, J. I. Criticality, the area law, and the computational power of projected entangled pair states. *Physical Review Letters* **96**, 220601 (2006).

[3] Gu, Z.-C., Levin, M., Swingle, B. & Wen, X.-G. Tensor-product representations for string-net condensed states. *Physical Review B* **79**, 085118 (2009).

[4] Deng, D.-L., Li, X. & Sarma, S. D. Exact machine learning topological states. Preprint at http://arxiv.org/abs/1609.09060 (2016).

[5] Arora, S. & Barak, B. *Computational complexity: a modern approach* (Cambridge University Press, 2009).

[6] Gao, X., Wang, S.-T. & Duan, L.-M. Quantum supremacy for simulating a translation-invariant ising spin model. *Physcal Review Letters* **118**, 040502 (2017).

[7] Broadbent, A., Fitzsimons, J. & Kashefi, E. Universal blind quantum computation. In *Proceedings of the 50th Annual IEEE Symposium on Foundations of Computer Science (FOCS 2009)*, 517–526 (IEEE, 2009).

[8] Babai, L., Fortnow, L. & Lund, C. Nondeterministic exponential time has two-prover interactive protocols. In *Proceedings of the 31th Annual IEEE Symposium on Foundations of Computer Science (FOCS 1990)*, 16–25 (IEEE, 1990).

[9] Boixo, S. *et al.* Characterizing quantum supremacy in near-term devices. Preprint at http://arxiv.org/abs/1608.00263 (2016).

[10] Le Roux, N. & Bengio, Y. Representational power of restricted boltzmann machines and deep belief networks. *Neural Computation* **20**, 1631–1649 (2008).

[11] Huang, Y. & Moore, J. E. Neural network representation of tensor network and chiral states. Preprint at http://arxiv.org/abs/1701.06246 (2017).

[12] Chen, X., Zeng, B., Gu, Z.-C., Chuang, I. L. & Wen, X.-G. Tensor product representation of a topological ordered phase: Necessary symmetry conditions. *Physical Review B* **82**, 165119 (2010).

[13] Schuch, N., Wolf, M. M., Verstraete, F. & Cirac, J. I. Computational complexity of projected entangled pair states. *Physical Review Letters* **98**, 140506 (2007).

[14] Barenco, A. *et al.* Elementary gates for quantum computation. *Physical review A* **52**, 3457 (1995).

[15] Nielsen, M. A. & Chuang, I. L. *Quantum computation and quantum information* (Cambridge university press, 2010).

[16] Carleo, G. & Troyer, M. Solving the quantum many-body problem with artificial neural networks. *Science* **355**, 602–606 (2017).

[17] Long, P. M. & Servedio, R. Restricted boltzmann machines are hard to approximately evaluate or simulate. In *Proceedings of the 27th International Conference on Machine Learning (ICML-10)*, 703–710 (2010).

[18] Koller, D. & Friedman, N. *Probabilistic graphical models: principles and techniques* (MIT press, 2009).

[19] Goodfellow, I., Bengio, Y. & Courville, A. *Deep learning* (MIT press, 2016).
